# Commentary: Deep learning in obsessive-compulsive disorder: a narrative review

**DOI:** 10.3389/fpsyt.2026.1776019

**Published:** 2026-02-17

**Authors:** Olga Kashevarova, Galina Portnova, Guzal Khayrullina, Krystsina Liaukovich, Ekaterina Proshina, Anastasia Gaidareva, Rohit Verma, Rupali Bhardwaj, Olga Martynova

**Affiliations:** 1 Laboratory of Human Higher Nervous Activity, Institute of Higher Nervous Activity and Neurophysiology of the Russian Academy of Sciences, Moscow, Russia; 2 Faculty of Biology and Biotechnology, National Research University Higher School of Economics, Moscow, Russia; 3 Department of Psychiatry, All India Institute of Medical Sciences, New Delhi, India; 4 Computer Science and Engineering Department, Thapar Institute of Engineering and Technology, Patiala, India

**Keywords:** deep learning, diagnostic prediction, neuroimaging, obsessive-compulsive disorder, precision psychiatry, treatment prediction

## Introduction

The narrative review by Zaboski et al. (1) provides a timely and valuable synthesis of the application of deep learning (DL) to obsessive-compulsive disorder (OCD). The authors effectively argue for the unique potential of DL to handle the marked heterogeneity and complexity of OCD, which often eludes traditional statistical methods. By focusing exclusively on OCD, the review offers a necessary and nuanced examination that broader psychiatric AI reviews might miss. The reported diagnostic accuracies of 80–98% are certainly impressive and validate the technical promise of these approaches. However, as the field stands at this promising juncture, the most critical challenge has shifted from proving technical feasibility to ensuring clinical translation. Building on this foundation, the present discussion highlights the challenges of scalability and clinical implementation while advocating for two specific research avenues: data-driven subtyping and genetic integration.

## Challenges in clinical translation

Zaboski et al. (1) correctly identify that models relying on resource-intensive data like functional magnetic resonance imaging (fMRI) or clinician-administered scales must demonstrate “incremental utility” beyond specialist diagnosis. While impressive performance metrics are achieved in controlled research settings, the “last mile” to the clinic is fraught with practical obstacles. For instance, the proof-of-concept by Wahl et al. (2) for detecting hand-washing via smartwatch is compelling, yet its real-world utility depends on seamless integration into patients’ lives and clinical practice. Issues of user compliance, device battery life, data synchronization, and false positive management present significant barriers to sustained use. Future research should focus on both model optimization and implementation feasibility.

Furthermore, the review’s discussion on leveraging electronic medical records (EMRs) for scalable detection is a vital direction. However, it is crucial to acknowledge that EMR data comes with its own set of complexities, including significant selection bias (over-representing severe cases), fragmented documentation across different healthcare providers, and a high degree of unstructured, “noisy” data. Developing models that are robust to these real-world data imperfections is a fundamental prerequisite for their clinical application.

## Data-driven subtyping and genetic integration

A significant opportunity beyond diagnostic classification, also highlighted in the review, lies in using DL to deconstruct the marked heterogeneity of OCD into empirically grounded, data-driven subtypes. Current subtyping based on symptom dimensions (e.g., contamination/washing, symmetry/ordering) has clinical utility but may not capture underlying neurobiological or genetic distinctions. Indeed, a growing body of evidence, including work from our group, suggests that distinct OCD subtypes are underpinned by divergent neurobiological pathways involving dysregulation across glutamatergic, serotonergic, dopaminergic, and neurotrophic systems, as well as distinct patterns of brain region engagement (3). This neurobiological heterogeneity provides a strong rationale for applying DL models, which are uniquely suited to analyze high-dimensional multimodal data, such as neuroimaging, clinical scales, and behavioral data from wearables, to identify clusters of patients that share common biological and symptom trajectories (4). For example, a model might discover a subtype characterized by specific cortico-striatal connectivity patterns, high autonomic arousal during compulsions, and poor response to anti-obsessive medications but better response to exposure and response prevention (ERP) therapy (5). A network-level dysregulation contributes to OCD pathophysiology (6) and highlights the importance of connectivity analyses in subtyping efforts. Such a data-driven taxonomy would advance precision medicine beyond a one-size-fits-all diagnostic label.

The review mentions OCD’s genetic basis but notes the scarcity of DL applications in this area. Integrating genetic data, such as polygenic risk scores or specific risk variants from genome-wide association studies, into multimodal DL models could powerfully enhance their predictive and explanatory power (7). A model that simultaneously processes a patient’s fMRI data, symptom profile, and genetic risk could achieve several goals: 1) identify individuals at high risk before the full onset of disorder, enabling preventive strategies; 2) uncover how genetic risk manifests in specific brain circuit dysfunctions; and 3) significantly improve the prediction of treatment response by linking genetic markers to pharmacological mechanisms. While this vision is compelling, its realization requires navigating substantial practical challenges. These include the need for rigorous data harmonization across heterogeneous genetic datasets, careful control for population stratification to avoid spurious findings, and the ongoing challenge of assembling the large, multimodal sample sizes required for robust DL analysis. While sample sizes for such integrated analyses remain challenging, consortia like ENIGMA-OCD and the International OCD Foundation Genetics Collaborative are building the necessary large-scale datasets to eventually overcome these hurdles.

## Explainability and mechanistic understanding

The authors briefly mention model interpretability, but this issue warrants center stage in future work. The “black box” nature of many DL models is a major barrier to clinician adoption and patient trust. A clinician is unlikely to base a high-stakes treatment decision on a model’s output without understanding the rationale, especially when contradicting clinical judgment.

The next wave of innovation must invest in Explainable AI (XAI) techniques tailored to psychiatric applications. For example, generating saliency maps that highlight which brain regions or genetic loci most influenced a classification or subtype assignment can build essential trust and provide clinically meaningful insights. Concretely, such outputs could be integrated into clinical workflows by visually overlaying a saliency map on a patient’s own brain scan during a case review, pinpointing circuits of high model relevance. This could directly inform the targeting of neuromodulation therapies or the psychoeducation of a patient about the neurobiological basis of their symptoms. Moreover, integrating behavioral and electrophysiological data can illuminate mechanisms underlying model predictions. The recent antisaccade studies revealed attentional bias toward negative stimuli in OCD, with corresponding electrophysiological abnormalities suggesting emotional dysregulation’s role in impaired inhibitory control (8, 9). Thus, more proactive selection of feature space can improve the model accuracy and increase their physiological interpretation.

Zaboski et al. (1) have clearly outlined the promising potential of DL in OCD research. The path forward requires a multifaceted approach that prioritizes several key objectives. First, DL must be leveraged to discover data-driven OCD subtypes grounded in neurobiological and genetic distinctions. Second, integrating genetic data into multimodal models could enable early identification of high-risk individuals before full disorder onset, uncover how genetic risk manifests in specific brain circuit dysfunctions, and improve treatment response prediction by linking genetic markers to pharmacological mechanisms. While acknowledging the practical constraints of data harmonization and sample size. Third, explainability must become central to model development, integrating behavioral and electrophysiological insights to build clinician trust and patient engagement, for instance by providing interpretable visual aids for clinical decision-making. Finally, prospective clinical trials are needed to validate these approaches and test real-world impact on patient outcomes. By addressing these translational challenges with the same rigor applied to algorithmic innovation, the field can ensure that DL’s remarkable potential translates into meaningful advancements in precision psychiatry for the millions affected by OCD.
